# Assessment of Silicon- and Mycorrhizae- Mediated Constitutive and Induced Systemic Resistance in Rice, *Oryza sativa* L., against the Fall Armyworm, *Spodoptera frugiperda* Smith

**DOI:** 10.3390/plants10102126

**Published:** 2021-10-07

**Authors:** Santhi Bhavanam, Michael J. Stout

**Affiliations:** Department of Entomology, Louisiana State University Agricultural Center, Baton Rouge, LA 70803, USA; MStout@agcenter.lsu.edu

**Keywords:** rice, silica, injury, *Glomus intraradices*, AMF, induction, defense priming, generalists, pest management

## Abstract

Induced resistance provides protection in plants against insect herbivores. Silicon and mycorrhizae often prime plant defenses and thereby enhance plant resistance against herbivores. In rice, *Oryza sativa* L., insect injury has been shown to induce resistance against future defoliators. However, it is unknown if silicon and mycorrhizae treatments in combination with insect injury result in greater induced resistance. Using the fall armyworm (FAW), *Spodoptera frugiperda* Smith, two experiments were conducted to investigate whether (1) silicon or mycorrhizae treatment alters resistance in rice and (2) induced systemic resistance in response to insect injury is augmented in silicon- or mycorrhizae- treated plants. In the first experiment, silicon treatment reduced FAW growth by 20% while mycorrhizae increased FAW growth by 8%. In the second experiment, insect injury induced systemic resistance, resulting in a 23% reduction in FAW larval weight gains on injured compared to uninjured plants, irrespective of treatment. Neither silicon nor mycorrhizae enhanced this systemic resistance in insect-injured plants. Furthermore, mycorrhizae resulted in the systemic increase of peroxidase (POD) and polyphenol oxidase (PPO) activities, and injury caused a slight decrease in these enzyme activities in mycorrhizae plants. Silicon treatment did not result in a stronger induction of POD and PPO activity in injured plants. Taken together, these results indicate a lack of silicon and mycorrhizae priming of plant defenses in rice. Regardless of injury, silicon reduced FAW weight gains by 36%. Based on these results, it appears silicon-mediated biomechanical rather than biochemical defenses may play a greater role in increased resistance against FAW in rice.

## 1. Introduction

Plants protect themselves from herbivores directly, through the expression of secondary metabolites and the formation of morphological structures such as trichomes and epicuticular waxes, or indirectly, through the emission of plant volatiles or the formation of structures that facilitate the activities of parasitoids and predators of herbivores [1]. Plant defenses can be constitutive, present irrespective of herbivory, or induced, expressed in response to external stimuli including herbivore infestation and pathogen infection [2,3]. Induced resistance can be observed not only at the site of infestation, which may have an impact on the pathogen or herbivore that caused the initial injury, but also systemically, in plant parts that are distant from injured plant tissues or even in plant parts that are not present at the time of infestation [2]. Induced systemic resistance against biotic stresses is regulated by several signaling pathways, including those mediated by salicylic (SA) and jasmonic (JA) acid [3]. The JA pathway is activated in response to chewing insects or necrotrophic pathogens, and the activation of this pathway often results in the production of plant secondary metabolites that are deterrent or toxic to insects and/or the release of plant volatiles that attract natural enemies of herbivores [4,5]. Induced resistance is an aspect of phenotypic plasticity and allows plant resistance to be expressed only when needed, thereby conserving plant resources that can be used for plant growth and reproduction [6]. Moreover, induced resistance allows plants to express defensive strategies that are appropriate to the type of abiotic and biotic stresses they encounter [6]. The existence of robust and specific induced resistance mechanisms in plants could be exploited for pest management [1] as it can reduce reliance on insecticides and aid in the development of sustainable pest management strategies. Several studies have demonstrated that the application of plant elicitors or infestations with insects or pathogens can induce resistance against herbivores of crop plants [1]. 

Many studies have investigated whether the application of silicon to soil or the inoculation of roots with arbuscular mycorrhizal fungi can enhance the resistance of plants against herbivory. More recently, some studies have also investigated whether these two treatments can prime plants to make them more responsive to herbivory [7,8]. Silicon is now considered a ‘beneficial nutrient’ because of its important role in enhancing plant growth [9] and the resistance of plants to biotic and abiotic stresses [9]. Silicon is absorbed by roots in the form of monosilicic acid, translocated through xylem vessels, and deposited in shoots and leaves as amorphous silica [10,11] in the form of biogenic opals (phytoliths) beneath cell walls, forming a physical barrier [12]. Silicon deposited in and around cells increases abrasiveness and mechanical toughness of plant tissues [12]. This biomechanical mode of action, which is widely considered the main form of silicon-mediated resistance against herbivores [13] can reduce the fitness of insects [14,15,16]. In addition, silicon can increase resistance in plants through the modulation of biochemical and molecular processes related to plant defense systems [13] because silicon can activate the JA pathway [7]. Silicon supplementation elevates the activities of plant antioxidants and resistance-related enzymes such as catalase (CAT), peroxidase (POD), polyphenol oxidase (PPO), and phenylalanine ammonia lyase (PAL) [7,17]. 

Arbuscular mycorrhizal fungi form symbiotic relationships with a wide range of plant species including agricultural crops [18]. Mycorrhizae increase the uptake of macro- and micro-nutrients by plants [18], thereby alleviating nutrient stress and increasing plant growth and fitness [18]. Moreover, mycorrhizae-associated changes in plant nutritional quality, biomass, vigor, and resistance-related compounds can make plants either more susceptible or resistant to herbivory and pathogens [19]. In general, specialists (insect species that feed on one or a few closely related plant taxa, often a single genus) perform better on plants inoculated with mycorrhizae [19] while generalists (insect species that feed on plant species belonging to more than one plant family), in particular chewing generalist insects, are negatively impacted by mycorrhizae [20,21], although a few studies have reported positive [22,23] or neutral effect [24] of mycorrhizae on generalists. The influence of mycorrhizae on plant and herbivore resistance is dependent on time since inoculation and level of colonization [25,26] because colonization and the establishment of mycorrhizae and associated changes in plants take time and hence the impact of mycorrhizae is often not evident until a few weeks post-inoculation [27,28]. Recent studies have shown that mycorrhizae can enhance resistance against insects and pathogens through the systemic activation of plant defenses, referred to as systemic resistance [29]. Moreover, activation of the JA pathway due to mycorrhizal inoculation can prime plants, with the result that plant defenses are expressed more quickly and strongly following herbivory and pathogen attacks [29]. A few studies have demonstrated stronger upregulation of plant defense genes, including the JA and phenylpropanoid pathways, in mycorrhizal plants relative to non-mycorrhizal plants upon insect infestation or pathogen infection [8,30]. However, a few other studies have reported the suppression [31,32] of plant defenses by mycorrhizae, indicating that the effects of mycorrhizae-induced resistance are variable. 

Rice, *Oryza sativa* L., is an important crop globally and in the southern United States. This species has long served as a model to study the effects of silicon amendment on plant growth and resistance against pests. Rice is an active accumulator of silicon and contains about 10% silicon by dry mass [33]. Studies have shown that supplementation of rice with silicon increased resistance against chewing and sucking insects. For example, Han, et al. [34] reported that a lepidopteran insect, the rice leaf folder *Cnaphalocrocis medinalis* Guenee, exhibited increased larval development time, increased larval mortality, and larval mass when fed on silicon-treated rice plants than when fed on silicon-untreated rice plants. Moreover, in rice, silicon amendment (0.32 g/kg soil) significantly reduced phloem ingestion of the brown planthopper, *Nilaparvata lugens* Stal, contributing to a decreased population growth rate and net reproductive rate [35]. More recently, rice has also served as a model to investigate the effects of mycorrhizal colonization on resistance to pests, with variable results. Cosme, et al. [36] showed that mycorrhizal rice plants were preferred by the rice water weevil (RWW), *Lissorhoptrus oryzophilus* Kuschel, for oviposition. Increased susceptibility in mycorrhizal-inoculated plants to rice water weevils, fall armyworm, (FAW), *Spodoptera frugiperda* Smith, and stem borers was also observed in the greenhouse and field plots [37,38]. In contrast, mycorrhizal inoculation enhanced resistance in rice plants against blast [39]. Variation among studies may be due to different mycorrhizae species used as mycorrhizae impact varies depending on the plant, mycorrhizae species, and pathogen/insect species tested. 

Stout, et al. [40] demonstrated that previous injury or application of an elicitor to young rice plants resulted in the expression of induced local and systemic resistance. The results of feeding bioassays conducted 4–5 days (local resistance) and 12 days (systemic resistance) after defoliation of young rice plants showed that the relative growth rates (RGR) of FAW were lower for larvae fed on leaves derived from injured plants compared to larvae fed on leaves obtained from uninjured plants and that the reductions in RGR were higher for induced systemic resistance compared to induced local resistance. To our knowledge, no further studies have investigated direct and systemic induced resistance to defoliating insects in rice alone or in combination with other treatments such as silicon and mycorrhizae. Furthermore, no attempts have been made to elucidate the mechanisms involved in this kind of induced systemic resistance in rice.

As summarized above, silicon, mycorrhizal colonization, and prior herbivory have all been shown to affect the resistance of rice independently. However, there have been no studies of the interactive effects of these three factors on the resistance of rice to herbivores. Therefore, the present study was undertaken to determine whether (1) silicon (Si), mycorrhizae (AMF), and silicon in combination with AMF increases (constitutive) resistance in rice plants against FAW; and (2) silicon, AMF, and silicon in combination with AMF prime plants respond more strongly to herbivory in rice.

## 2. Results

### 2.1. Feeding Bioassays

**Figure 1:**
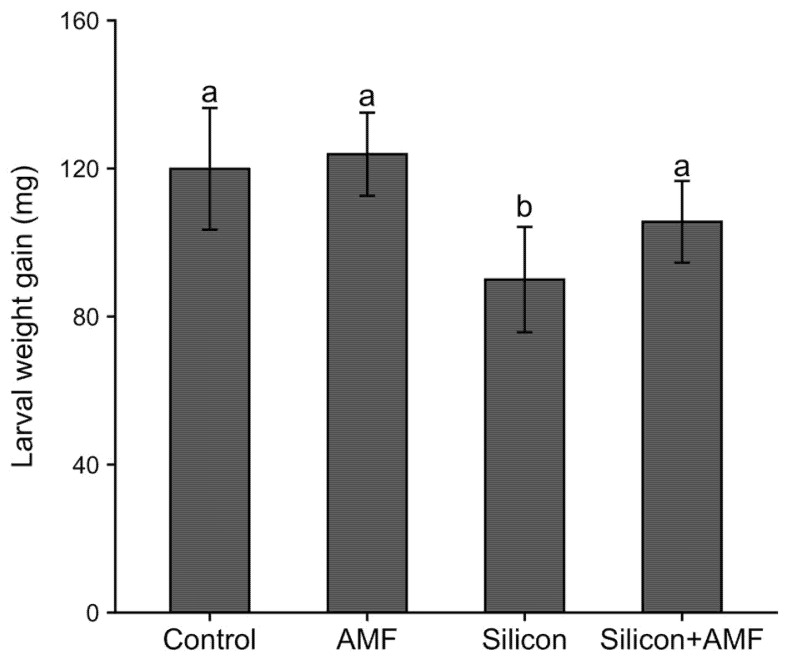
Mean three-day larval weight gains (mg ± S.E.) of fall armyworm, *Spodoptera frugiperda*, fed on leaves derived from rice, *Oryza sativa*, grown in soil treated either with silicon (Si^+^AMF^−^), mycorrhizae (AMF; Si^−^AMF^+^), silicon plus AMF (Si^+^AMF^+^) or untreated control (Si^−^AMF^−^). Error bars indicate standard error. Different lowercase letter above each bar represent means that differed significantly.

Larvae fed on plants treated with silicon gained less weight compared to larvae fed on plants not treated with silicon (F_1,59_ = 14.08, *p* = 0.000) (Figure 1). The reduction in weight gain in silicon-treated plants relative to untreated plants was about 20%. Additionally, the main effect of mycorrhizae inoculation had an overall significant positive impact on FAW growth (F_1,59_ = 7.73, *p* = 0.007), although Tukey’s *posthoc* comparisons failed to find differences among control and mycorrhizae-treated plants. Larval weight gains increased by 8% on mycorrhizae plants compared to non-mycorrhizae plants (Figure 1). There was no significant interaction between silicon and mycorrhizae on weight gains of FAW larvae (F_1,59_ = 1.89, *p* = 0.175). 

**Figure 2:**
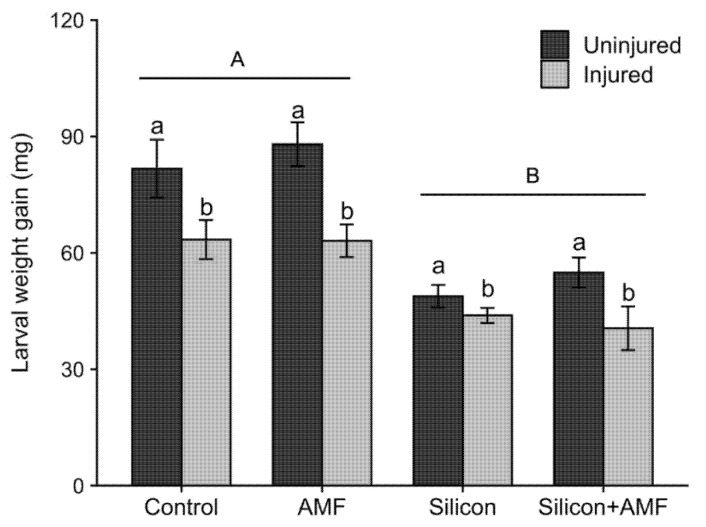
Mean (±SE) larval weight gains of the fall armyworm, *Spodoptera frugiperda*, fed on new leaves obtained from FAW-injured (grey bars) or corresponding new leaves from uninjured (black bars) rice, *Oryza sativa*, plants that were treated with silicon (Si^+^AMF^−^), mycorrhizae (AMF; Si^−^AMF^+^), silicon plus AMF (Si^+^AMF^+^), or control (Si^−^AMF^−^). Error bars represent standard errors. Uppercase letters above horizontal bars represent significant differences in larval weight gains fed on silicon-treated and untreated plants (*p* < 0.05). Within each soil treatment, lowercase letters above vertical bars indicate significant differences (*p* < 0.05).

The second set of feeding bioassays that investigated whether Si, AMF, injury, and their interactions induce systemic resistance against FAW in rice showed that only the main effects of Si and injury had a significant impact on FAW larval weight gains (Table 1). Regardless of soil treatment, FAW larvae fed on new leaves produced on plants that were injured 12 days before the bioassay gained significantly less weight compared to those fed on corresponding new leaves taken from uninjured plants, indicating that injury led to induced systemic resistance in rice (Table 1; Figure 2). The percent reductions in weight gains were about 23% on injured plants compared to uninjured plants. Larval weight gains were also significantly affected by silicon application; weight gains of FAW larvae were on average 36% lower on plants supplied with silicon than silicon untreated plants (Table 1; Figure 2). Additionally, the interaction between silicon and injury had a marginally significant effect on larval weight gains, probably because the induced resistance was weaker in plants treated with silicon (Table 1). In these feeding bioassays, unlike the first set, AMF inoculation did not have a significant effect on larval weight gain (Table 1; Figure 2). Moreover, the interaction of AMF and injury had no effect on FAW growth (Table 1). Furthermore, the three-way interaction between Si, AMF, and injury did not have a significant effect on FAW larval weight gain (Table 1). 

### 2.2. Plant Phenolics Content, Peroxidase, and Polyphenol Oxidase Activities

**Figure 3:**
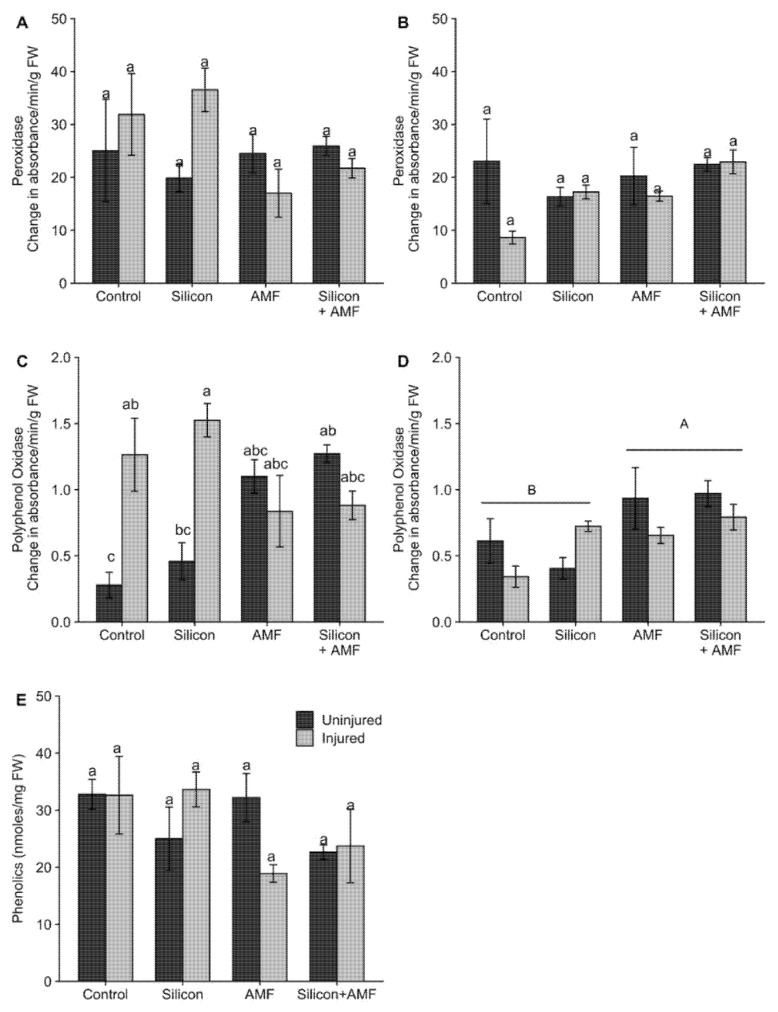
Activities of peroxidase at 72 h (**A**) and 12 d (**B**), polyphenol oxidase at 72 h (**C**), and 12 d (**D**) and phenolics content at 48 h (**E**) in uninjured (black bars) and injured (grey bars) rice, *Oryza sativa* plants were treated either with silicon (Si^+^AMF^−^), mycorrhizae (AMF; Si^−^AMF^+^), silicon plus AMF (Si^+^AMF^+^), or untreated control (Si^−^AMF^−^). Phenolics content is expressed as nmoles ferulic acid equivalents per mg of fresh weight of leaf tissue. Peroxidase and polyphenol oxidase activities are reported as change in absorbance/min/g of fresh weight of leaf tissue. Values are mean ± SE. In each figure different lowercase letters above bars indicate significant differences in means (*p* < 0.05). Uppercase letters above bars indicate significant differences in PPO activity at 12 d after injury between mycorrhizae inoculated and uninoculated plants. Error bars indicate standard error. Number of replicates = 3.

The main effects of injury and the interaction of injury and mycorrhizae had a significant effect on activity of PPO at 72 h after injury (Table 2; Figure 3C). Activities of PPO were substantially lower in injured mycorrhizae plants relative to injured non-mycorrhizae plants while in uninjured plants, mycorrhizae inoculation significantly increased activities of PPO compared to non-mycorrhizae plants. At 72 h after injury, only the mycorrhizae by injury interaction was significant for POD activity (Table 2; Figure 3A). However, *posthoc* analysis revealed no significant differences among treatments (Figure 3A). At 12 d after injury, only the main effect of AMF had a significant effect on PPO activity (Table 2, Figure 3D). Overall, PPO activities were higher in plants treated with AMF compared to other treatments. Neither the main effects of silicon, AMF, and injury nor the interactions between these main effects had a significant influence on phenolics contents at 48 h after injury (Table 2; Figure 3E) or POD activity at 12 d after injury (Table 2, Figure 3B). 

## 3. Discussion

The first hypothesis tested in this study was that amending soil with silicon or inoculating soil with mycorrhizae would directly alter the resistance of rice plants to FAW larvae. Larvae fed on plants supplied with silicon gained 20% less weight compared to larvae fed on control plants (Figure 1). This result is consistent with numerous other studies showing that the application of silicon, either through foliar spray or soil amendment, reduces the growth, development, and reproduction of insects [14,41]. In rice, silicon supplementation increases silicon content [17]. Thus, the decreases in FAW growth on silicon-amended plants in this study may be related to the deposition of silicon in the leaves, leaf sheaths, and culms of rice plants. Silicon deposition causes mandible wear and limits larval feeding. This mechanism reduced the larval growth of the African armyworm, *Spodoptera exampta* Walker, feeding on silicon-treated grasses [42]. Moreover, the accumulation of silicon in trichomes on the leaf surface and between cells increases leaf abrasiveness, which may damage insect midgut epithelia as was observed for the tomato leafminer, *Tuta absoluta* Meyrick [43], and reduce the digestibility of plant tissues and nutrient absorption by insects. Furthermore, Hunt, et al. [44] showed that the presence of silicon between plant cells increased cell toughness that prevented cell breakdown and the release of nutrients in the insect gut, resulting in reduced food conversion efficiency in the desert locust, *Schistocerca gregaria* Forskal. 

This study also found that the inoculation of rice plants with a mycorrhizal species, *R. intraradices*, had a slight but significant positive effect on FAW growth. Overall, larvae gained 8% more weight on mycorrhizae plants compared to non-mycorrhizae plants (Figure 1). In rice, previously, it was shown that mycorrhizae had a positive impact on rice herbivory: FAW larvae fed for four days on rice plants treated with a mixture of six AMF species gained approximately 30% more weight than larvae fed on uninoculated plants [37], consistent with other studies that reported better performance of herbivores on plants inoculated with a mixture of AMF species [19,21]. Similarly, Cosme, et al. [36] found that an oligophagous insect, the rice water weevil, preferred and laid more eggs on 14- to 18-day-old rice plants inoculated with *G. intraradices* relative to non-mycorrhizae plants, in accordance with general findings that specialists, compared to generalists, perform better on mycorrhizae plants [19]. However, a meta-analysis of 34 studies found that *G. intraradices* inoculation often reduces insect growth, especially generalists [19]. For example, Selvaraj, et al. [20] reported that tobacco cutworm, *Spodoptera litura* F., larvae fed on *G. intraradices*-treated black gram, *Vigna mungo* L., plants had lower leaf consumption rates and food conversion efficiencies, which resulted in decreased RGR relative to non-mycorrhizae plants. Our results are in contrast with this general pattern that *G. intraradices* has a negative impact on the performance of generalist chewing insects.

The effects of mycorrhizae on insect growth often vary with host plants, mycorrhizae species, and feeding specializations, among other factors. Although it is unclear why *G. intraradices* increased FAW growth in this study, this AMF effect may have been related to the stage of the mycorrhizal colonization process, as indicated by time post-inoculation and the level of colonization. These two factors can determine mycorrhizae-associated alterations in plant growth, nutrient quality, and defense chemistry and hence the impact of mycorrhizae on herbivory. For example, in soybean, *Glycine max* L., growth of the corn earworm, *Helicoverpa zea* Boddie and FAW was negatively correlated with levels of mycorrhizae colonization; with increases in colonization levels, larval developmental times increased and growth rates decreased in both species [45]. Likewise, in broad beans, *Vicia faba* L., aphid development was faster on plants inoculated with mycorrhizae seven weeks prior compared to development on plants that were inoculated with mycorrhizae at the same time as the aphid infestation. The percentage of root colonization on the former and the latter averaged 50% and 30%, respectively [46]. Our study did not quantify mycorrhizae colonization, but previous studies showed that *G. intraradices* can form symbiotic associations with several rice varieties under aerobic conditions [28,36,47]. In two previous studies on rice, the percentage of root colonization by *G. intraradices* increased over time from 5% at 30 days post-inoculation (dpi) to 25–30% at 45 dpi, with all stages of *G. intraradices,* including intraradical hyphae, arbuscules, and vesicles, appearing on the roots at 42 dpi [28,39]. Consequently, a positive impact of AMF inoculation on plant growth was evident at 42 dpi but not at 35 dpi when AMF colonization was low [28]. In the present study, rice plants were grown under unflooded conditions and watered when needed, so irrigation probably had no impact on mycorrhizae establishment. Given the short time period (21 days) from mycorrhizal inoculation to the start of feeding bioassays in our study, it is possible that the level of mycorrhizal colonization was still low, resulting in a slight positive effect of mycorrhizae on FAW growth. However, it is predicted that this effect of mycorrhizae on FAW growth may have shifted towards negative or neutral with progress in root colonization and establishment. Alternatively, during the initial stages of mycorrhizae establishment, the expression of defense signaling pathways often shifts [39,48], making the rice plants susceptible to herbivores.

The second hypothesis tested in this study was that amending soil with silicon or inoculating soil with mycorrhizae would alter the responsiveness of rice to injury from feeding by FAW larvae (“priming”). In a previous report, Stout, et al. [40] established that feeding by FAW on rice induces resistance in leaves that develop 10–14 d after injury. The current study confirmed this prior result by showing that FAW larvae fed on leaves obtained from plants injured 12 days prior by FAW attained lower weight gains than larvae fed on uninjured plants (Figure 2). There was, however, no evidence that silicon treatment had a priming effect on plants. The marginally significant interaction between the silicon treatment and injury (Table 1) probably reflects a *lower* reduction in larval weight gains due to injury in silicon-treated plants and silicon-untreated plants (18% and 26%, respectively). As in the first set of experiments, silicon amendment reduced overall weight gains by about 36%. The increases in resistance due to silicon may have masked increases in resistance resulting from prior feeding. 

Silicon accumulation is considered to be a constitutive physical defense trait [41]. Hall, et al. [49] suggested that if silicon treatment already results in the expression of constitutive silicon-based plant defenses against herbivores, then a stronger induction of JA chemical defenses may not be required. Recently Johnson, et al. [50] and Hall, et al. [49] demonstrated that herbivory or the application of elicitors resulted in an increase in endogenous JA levels; however, this increase in JA levels was no higher or even lower in silicon-treated plants than untreated plants. In support of this, we showed that injury increased the activities of PPO in both injured silicon-treated plants and injured plants not treated with silicon at 72 h after injury, but no significant differences between the two groups of plants were detected. However, Kim, et al. [51] and Ye, et al. [7] reported that silicon priming of defenses resulted in a stronger expression of POD and PPO in silicon-treated rice plants exposed to the rice leaf folder. 

Besides silicon mediated induced chemical defenses, studies have shown that silicon absorption and accumulation itself is an inducible defense [52,53], and feeding by insects even for a short period significantly increased the uptake of silicon by plants [50]. This increased deposition induced either by elicitor application or herbivory conferred resistance not only in high silicon accumulators such as grasses but also in moderate silicon accumulators like cucumber and soybean [53]. For example, in the grass species *Brachypodium distachyon,* herbivory enhanced silicon uptake that led to an increased accumulation of silicon in leaf macro hairs, resulting in decreases in the RGR of the cotton bollworm, *Helicoverpa armigera* [50]. In cucumbers, silicon-induced responses resulted in a higher foliar silicon content and reduced larval relative consumption rates and RGR of the cotton bollworm [41]. In rice, the uptake, translocation, and deposition of silicon have been well-documented [54]. Rice is a high accumulator of silicon and absorbs silicon in the form of silicic acid actively by an influx, *Low silicon rice 1* (*Lsi1*) and an efflux, *Low silicon rice 2* (*Lsi2*) transporter [54]. The transporter *Lsi1* is involved in the movement of silicon from outside into root cells; silicon is then transported to the apoplasts by *Lsi2* [54] and subsequently to other plant parts. The expression of these transporters was altered in response to insect injury, resulting in higher absorption of silicon [7]. Although this study did not quantify silicon content in rice plants before and after injury, it is speculated that silicon accumulation might have increased after injury, which may have contributed to reductions of FAW weight gains on injured silicon plants in addition to at least some resistance provided by enhanced plant defense enzyme activities. The relative importance of the biochemical and biomechanical forms of resistance in silicon-treated plants needs further investigation.

In this study, induced resistance conferred by insect injury was not further amplified by *G. intraradices* inoculation in rice, a fact supported by the non-significant interaction between mycorrhizae and injury (Table 1; Figure 2). In fact, activities of PPO and POD after 72 h of insect injury trended lower in injured mycorrhizae-treated plants than in mycorrhizae-treated uninjured plants. Despite this decreased enzyme activity, FAW weight gains did not differ when fed on injured mycorrhizae plants and injured non-mycorrhizae plants (Figure 2). These results indicate that mycorrhizae did not prime for a stronger activation of plant defenses, at least for the expression of POD, PPO, and total phenolics in four-leaf stage rice plants (Table 2). Neither did mycorrhizal treatment interfere or suppress the induced resistance caused by insect injury in rice. In addition, we found that activities of PPO (and, to a lesser extent, POD) were higher in uninjured mycorrhizae-treated plants, indicating that mycorrhizae colonization might have resulted in the systemic activation and constitutive expression of POD and PPO in rice. Higher enzyme activity in mycorrhizae plants may be linked to the mycorrhizae colonization process as shown by Campos-Soriano, et al. [28] in rice.

It is thought that increased herbivore performance on mycorrhizae plants is sometimes associated with the enhancement of host plant nutrient quality or plant vigor/biomass in plants colonized by mycorrhizae [19]. Defoliation by chewing insects results in the removal of photosynthetic leaf material and lowers photosynthetic rates. Reduced photosynthetic capacity due to insect damage may limit the production and subsequent availability of resources in injured plants, which may have a neutral or negative effect on plant growth/vigor and foliar nutrient content, at least for a short period, and thereby offset the positive impact of mycorrhizae on the plant and thus on insect growth. In support of this, a study conducted by Kempel, et al. [23] on mycorrhiza-induced resistance using whole plant bioassays found grasses and dicots that were colonized by *G.* *intraradices* had greater biomass, which correlated with higher larval growth rates of the African cotton leafworm, *Spodoptera littoralis* Boisduval. However, in herbivory-injured plants, mycorrhizae failed to enhance plant biomass, and larvae fed on injured but *G. intraradices* -treated plants achieved a larval mass similar to larvae fed on induced but uninoculated plants, which was attributed partly to the diversion of resources away from growth in induced plants. The possibility that increased plant biomass has a positive impact on FAW growth is less likely in this study because (1) herbivory treatments were initiated when rice plants were 21 days old and previous studies on rice have shown that *G. intraradices* colonization reduced plant dry mass in rice plants less than 30 days old [28,36] and (2) feeding bioassays were conducted with excised leaf material supplied *ad libitum* in Petri dishes and during the course of the bioassays, and FAW larvae were not limited by available plant material. Additionally, Cosme, et al. [36] showed that foliar N and P contents were higher in mycorrhizae-treated rice plants relative to non-mycorrhizae plants. This study did not determine the changes in foliar nutrient content due to mycorrhizae but based on Cosme, et al. [36], it was assumed that the increased amount of nutrients in mycorrhizae-treated plants after injury may have been redirected for defense or storage for regrowth, resulting in the neutral effect of AMF on FAW growth after injury. Enhanced plant nutrient quality might also explain the small increase (8%) in FAW growth observed in uninjured mycorrhizae plants. However, this explanation is only tentative because shifts in phytohormonal signaling during the establishment of plant-mycorrhizae symbioses are still not well understood. It has been shown that defense signaling pathways, including JA and SA pathways, are sometimes altered to facilitate the establishment of mycorrhizae [29,48]. Mycorrhizae colonization may need to be well established for mycorrhizae to induce resistance or prime plants [29]. Previously, in rice, the effects of mycorrhizae colonization on plant defenses and induced resistance to pathogens have been investigated by Campos-Soriano, et al. [39]. They reported enhanced resistance against blast disease due to both systemic activation of plant defenses and priming of defense pathways in *G. intraradices*-inoculated rice plants at 40 dpi [39]. This discrepancy in results between Campos-Soriano, et al. [39] and the current study may be related to, as noted above, differences in the days post-mycorrhizae inoculation and percentage of root colonization, which may influence the expression of mycorrhizae-induced resistance or susceptibility in rice and in turn influence herbivore performance as demonstrated in other studies [24,25,55,56]. 

Studies of other plants have reported the accelerated and strengthened expression of plant defenses due to a priming effect by mycorrhizae. Schoenherr, et al. [8] reported the faster upregulation of plant defense genes and signaling pathways in mycorrhizae-treated relative to untreated potato, *Solanum tuberosum* L., plants that resulted in reduced larval weight gain by the cabbage lopper, *Trichoplusia ni* Hubner. Likewise, Song, et al. [30], using tomato, *Solanum lycopersicum* Mill., wild type, and mutant lines, showed mycorrhizae-increased resistance against cotton bollworm was due to the systemic priming of the JA pathway and defenses. Minton, et al. [57] showed that mycorrhizae, in combination with JA, induced activities of some (POD and proteinase inhibitor) but not all (PPO) plant chemical defenses, but these induced changes did not confer resistance in plants against *Manduca sexta* in nightshades, *Solanum* spp. 

Mycorrhizae may not always prime plant defenses; instead, they sometimes activate plant defenses systemically, leading to the constitutive expression of plant secondary metabolites that increase protection against herbivores. For instance, in black gram, mycorrhizae colonization resulted in the increased activities of PPO, POD, and PAL that correlated with lower feeding rates and decreased relative growth rates of tobacco cutworm [20]. In barrel medic, *Plantago lanceolate*, plants, mycorrhizae inoculation induced the production of two plant defense compounds, catalpol, and aucubin. Constitutive levels of the former compound were significant, and levels of the latter were substantially higher in mycorrhizae plants compared to non-mycorrhizae plants. Although herbivory did not further induce these compounds in mycorrhizae plants, differences in the relative abundance of these two compounds contributed to reductions in RGR of beet armyworm larvae [58]. In contrast, a few studies have shown a reduced expression of defenses in mycorrhizae plants that make the plants susceptible to herbivores [31,32,56]. These variable AMF effects are due to differences in the host plant, AMF species, herbivore, root colonization, and environmental conditions.

## 4. Conclusions

Our study shows that in rice, feeding by FAW larvae induces the systemic production of plant chemical defenses such that FAW larvae fed on the new growth of injured plants gained relatively less larval weight. Silicon amendment increased resistance in rice plants against FAW. However, it appears that the greater effect of silicon on FAW stems from silicon enhancement of constitutive resistance rather than potentiation of induced resistance. In addition, we found that silicon amendment did not result in priming for the stronger activation of plant defense (PPO) and antioxidant (POD) enzymes measured at 72 h after injury. FAW larvae fed on young rice plants grown in soil inoculated with AMF gained more weight. However, this positive effect of AMF on insect growth was not observed on mycorrhizae plants subjected to insect injury. Moreover, AMF inoculation resulted in the systemic activation of PPO and POD, but enzyme activities decreased slightly after injury, indicating a lack of a mycorrhizae priming effect. The absence of a strong effect of AMF colonization on insect growth may be because AMF was still in the early stages of establishment. It is hypothesized that, as the plant ages, AMF might increase herbivore growth before induction but not after induction and AMF may have a negative effect on herbivory at later stages of plant growth.

## 5. Materials and Methods

### 5.1. Insects

Larvae of the FAW were used both to induce resistance and to assess resistance in these experiments. The FAW is a sporadic pest of rice in the southern U.S. and Louisiana, mostly infesting young rice before a permanent flood is established. The source of insects used in this study was a colony of FAW maintained in a lab at Louisiana State University (LSU), Baton Rouge, LA. The colony was initiated with larvae collected in and around rice plots, Crowley, LA. Larvae were reared on a meridic diet (Stonefly Heliothis diet, Ward’s Science, Rochester, NY, USA) in 30 mL plastic cups (Dart Conex Complements Portion Cups, Plantation, FL, USA) in an environmentally-controlled room kept at 27 °C under a 14 h light: 10 h dark photoperiod. Pupae were collected and placed in plastic buckets that were lined with an oviposition substrate and covered with a cheesecloth. After eclosion, adults mated and oviposited in plastic buckets. Adults were provided with water and honey. Eggs were collected from the buckets and placed in 8-cell trays (Bio-Serv, Flemington, NJ, USA) until hatching. Newly hatched neonates were carefully placed on the diet in plastic cups. Two larvae were reared per cup. Larvae needed for bioassays were taken from this colony.

### 5.2. Plants

Seeds of the rice cultivar ‘Cheniere’ used for this study were obtained from the H. Rouse Caffey Rice Research Station, Crowley, LA. Cheniere is a widely grown, long-grain, early maturing rice variety that is susceptible to insect pests. Rice plants were grown in round pots (14 cm top diameter × 11.5 cm depth × 10 cm bottom diameter) containing a soil mix composed of 2 parts topsoil, 1 part sand, and 1 part peat in a greenhouse facility located near LSU, Baton Rouge, LA. Prior to planting, one of the four soil treatments described below was administered to soil in each pot. Five rice seeds were sown in each pot. Seven days after planting (dap), plants were thinned to four plants per pot. After thinning, plants were supplied with 1.5 g of 13-13-13 controlled-release fertilizer (Carlpool products, Galveston, TX, USA). The plants were watered as needed and grown on a bench in a greenhouse at a temperature ranging between 25–30 °C under ambient lighting.

### 5.3. Treatments

Treatments were prepared and administered to soil in pots before sowing rice seeds. Treatments were as follows: (1) control (Si^−^AMF^−^); (2) AMF (Si^−^AMF^+^); (3) Si (Si^+^AMF^−^); and (4) Si plus AMF (Si^+^AMF^+^). The mycorrhizal inoculum was a commercial product purchased from Wallace Organic Wonder, Premium Mycorrhizal Inoculant, Greene, RI, USA. The inoculum consisted of a single species, *Rhizophagus (Glomus) intraradices* (Walker & Schubler) and was applied at label rate. In each pot, 10 g of AMF was mixed thoroughly into the top one-third of the soil. For the silicon treatment, silicon at the rate of 1.8 g per pot, a dose equivalent to 1120 kg Si per ha, was mixed into the top few cm of soil. Silicon was applied in the form of wollastonite (24% Si, Vansil W-10, Vanderbilt Minerals LLC, Norwalk, CT, USA). Finally, for the combination treatment, 1.8 g of Si and 10 g of AMF were mixed into the top few cm of soil. 

### 5.4. Feeding Bioassays

Two sets of feeding bioassays (each set consisting of two independent experiments) were conducted to investigate the two hypotheses of this study. The first set of bioassays was conducted to determine whether application of Si, AMF, or Si in combination with AMF increases resistance (constitutive) in rice plants against FAW. Ten pots were prepared for each soil treatment. Bioassays were initiated when rice plants possessed four leaves prior to tillering. From each pot of treatment, two plants were excised with scissors at soil level, cut into approximately 4 cm-long sections, and placed in a 9-cm Petri dish lined with moistened Whatman No. 1 filter paper (VWR International LLC, Radnor, PA, USA). The Petri dishes were closed with a lid and transferred from the greenhouse facility to a lab on ice. Prior to the start of the feeding bioassays, newly molted (<12 h) third instar (larval developmental stage) FAW were taken from the colony and placed individually in clean 1 oz plastic cups. After 3–4 h of starvation, each larva was individually weighed on a precision balance (Mettler–Toledo, *XS105* Dual Range, Columbus, OH, USA) and its initial weight was recorded. A single larva was placed in each petri dish containing plant material of one of the four treatments (Si, AMF, Si plus AMF, or control). The Petri dishes were sealed with parafilm (VWR International LLC, Radnor, PA, USA). Larvae were left to feed on plant material for three days in an environmentally-controlled room kept at 27 ºC under a 14 h:10 h (L:D) photoperiod. After 36 h, old plant material was replaced with new plant material of the same treatment. After 3 days, bioassays were terminated. Larvae were again starved for 3–4 h before final weight was recorded. The experiment was repeated two times, with eight replicates (Petri dishes) per treatment per experiment. Larval weight gains were calculated as the difference between final larval weight and initial larval weight. The larval weight gains were used for data analysis. 

A second set of feeding bioassays was undertaken to determine whether treatment of plants with Si, AMF, or Si plus AMF augments or strengthens the systemic resistance induced by FAW feeding [40]. The experimental design followed a 2 × 2 × 2 factorial scheme that included Si (treated or untreated) or AMF (uninoculated or inoculated) made at planting and injury (injured and uninjured) imposed at the 4-leaf stage. Treatments and plants were grown as described above and 20 pots were prepared for each soil treatment. When rice plants were at the 4-leaf stage, the twenty pots of each soil treatment were divided into two sets of 10 each. One set of plants of each soil treatment was injured by confining FAW to plants. To confine FAW larvae on plants, the four rice plants in each pot were enclosed in a clear plastic cylinder (8.5 cm diameter × 23 cm height) by inserting one end of the cylinder into the soil mix and closing the other end with a lid. Cylinders possessed mesh-covered holes to allow air movement. Four fourth-instar FAW (one larva per plant) were released into cylinders and removed from plants between 12–16 h after release. By this time, the amount of injury (defoliation) by FAW ranged between 75–90% in most pots. Plastic cylinders and larvae were removed from pots, and plants were left to grow on the greenhouse bench. In injured plants, plant growth was slow and uneven and as a result, some plants developed a new leaf relatively faster than others. On average, FAW-injured plants took approximately 12 days to produce a fully expanded new leaf. 

The bioassays were initiated with the new (recently emerged) leaf that developed after injury by FAW in the injured sets of plants and the corresponding newly expanded leaf in uninjured plants. From each treatment, the new leaf of each plant was excised with scissors. To ensure that larvae were not food-limited during bioassays and that all replicates received an equal amount of food, new leaves harvested from each plant of a single treatment were pooled, cut into 4 cm-long leaf sections, equally divided, and placed in separate Petri dishes lined with moist filter paper. Thereafter, to each petri dish a single freshly molted third instar FAW larvae that had been starved for 3–4 h and pre-weighed on a precision balance was assigned. Larvae were left to feed on the treated leaf material at 27 °C, 14 h L: 10 h D photoperiod for three days, after which time bioassays were terminated. Again, larvae were starved for 3–4 h before their final weight was recorded. Larval weight gains were calculated as described above. The entire experiment was repeated twice and, in each repetition, the sample size ranged between 5–7 in each treatment combination. 

### 5.5. Determination of Plant Defense Enzymes and Plant Phenolics

Using a third set of plants, changes in levels or activities of phenolics, POD, and PPO in response to FAW injury in plants treated either with Si, AMF, or a combination of Si and AMF were assessed. The same procedure used to injure young rice plants as described in Section 5.4 was used. At 48 h, 72 h, and 12 d after injury by FAW larvae, both injured and uninjured plants were collected and stored at −20 °C until future analysis. Leaf material without stems was used for the quantification of total phenolics, POD, and PPO activity. 

Total phenolics were quantified using the Folin–Ciocalteu method. Phenolics were measured after 48 h of injury. From each plant of each treatment combination, second and third leaves were excised, cut into small sections, and weighed. Then, leaf material was soaked in 5 mL of 50% methyl alcohol in 20 mL scintillation vials for extraction of phenolics. After 10 days, 500 µL of extract was added to a test tube, followed by distilled water (2.25 mL) and 500 µL of Folin–Ciocalteu reagent (Lot No # S1350, MP Biomedicals LLC, Solon, OH, USA) in sequence. The sample was vortexed and incubated for 5 min. Then to the sample, 500 µL of 20% Na_2_CO_3_ (Lot No # 19C0556834, VWR International LLC, Radnor, PA, USA) was added, vortexed, and incubated for 10 min. The absorbance was measured at 720 nm with a UV/Visible double beam spectrophotometer (UV-6300PC, VWR International LLC, Radnor, PA, USA). A standard curve was prepared using ferulic acid and total phenolics in the leaf samples, expressed as nmoles ferulic acid equivalents per milligram of fresh weight of leaf tissue. For each treatment combination, three replicates (leaf material from 1 plant = 1 replicate) were performed.

Peroxidase and polyphenol oxidase activities were measured at two time points: 72 h and 12 d after injury. For the 72 h time point, first and fourth leaves, and for the 12 d time point, the new leaf developed after injury in both injured and uninjured rice plants of each treatment were used to analyze activity of POD and PPO. Rice leaves were cut into small sections and weighed. Leaf samples were initially ground in liquid nitrogen and then in 3 mL potassium phosphate buffer (0.05 M; pH 6.7) that contained 1% polyvinylpolypyrrolidone (Lot No # BCBK1891V, Fluka Analytical, St. Louis, MO, USA) and 400 µL of 10% Triton-X (Lot No # 031M0301V, Sigma-Aldrich, St. Louis, MO, USA) with an ice-cold mortar and pestle. After grinding, the sample was transferred to a centrifuge tube, vortexed, and centrifuged for 10 min at 11,000× *g* at 4 °C. The supernatant was used to determine the activities of POD and PPO. To assess POD activity, 200 µL of enzyme extract was added to 1 mL of a substrate solution consisting of 7.2 mM guaiacol (Lot No # MKBH1979V, Sigma-Aldrich, St. Louis, MO, USA) and 12 mM H_2_O_2_ (Lot No # MKBF3446, Sigma-Aldrich, St. Louis, MO, USA) substrate. The change in absorbance was measured with a UV/Visible double beam spectrophotometer at 470 nm in kinetic mode. Activity of PPO was assayed according to published protocols [7,59] with slight modifications. Enzyme extract (500 µL) was added to 1 mL of 0.15 mM catechol (Lot No # 081M0020V, Sigma-Aldrich, St. Louis, MO, USA) substrate and 500 µL of potassium phosphate buffer. The change in absorbance was measured with a UV/Visible double beam spectrophotometer at 420 nm in kinetic mode. Activities of POD and PPO were expressed as changes in absorbance/min/g of fresh weight of leaf tissue [60,61]. Three replicates (with each replicate comprising leaf material from one plant) were used to analyze POD and PPO activity at each time point.

### 5.6. Statistics

Data analyses were performed in RStudio version 1.4 [62]. Larval weight gains were log-transformed prior to analysis to meet the assumptions of normality. A two-way ANOVA was used to test the effects of Si, AMF, and their interaction on FAW larval weight gain followed by Tukey’s post hoc analysis for means comparisons. Experiment was entered into the analysis as a random effect. To assess the effects of induced systemic resistance on FAW larval weight gains, a three-way ANOVA was performed with experiment as a random effect and Si (treated or untreated), AMF (inoculated or uninoculated), injury (uninjured or injured), and their interactions included as fixed factors. Likewise, three-way ANOVA was used to test the effects of Si, AMF, injury, and their interactions on levels of phenolics and activities of POD and PPO. Data on POD and PPO measured at two different timepoints were analyzed separately. Tukey’s test was used for post hoc means comparisons. Data of POD at 12 d after injury was non-normal for residuals and subjected to square-root transformation prior to analysis. Data were analyzed using ‘lmer’ function in ‘lmer4′ [63], ‘emmeans’ [64], and ‘multcomp’ [65] packages. Packages ‘cowplot’ [66] and ‘ggplot2′ [67] were used to prepare graphs. 

## Figures and Tables

**Table 1:** Summary of results of three-way ANOVA analyses of the effects of silicon (Si), mycorrhizae (AMF), injury, and their interaction on the fall armyworm, *Spodoptera frugiperda,* larval weight gains in rice, *Oryza sativa*.

Effect	df	F	*p*
Si	1, 94	80.74	**<0.001**
AMF	1, 94	0.48	0.488
Injury	1, 94	26.76	**<0.001**
Si * AMF	1, 94	0.04	0.836
Si * Injury	1, 94	3.82	0.054
AMF * Injury	1, 94	1.17	0.281
Si * AMF * Injury	1, 94	0.07	0.786

Bold *p* values indicate significant main/interaction effect. * indicates interaction between the main effects.

**Table 2:** Results of the three-way ANOVA analysis of main effects of treatment silicon (Si), mycorrhizae (AMF), injury and their interaction on phenolics contents and activities of peroxidase (POD) and polyphenol oxidase (PPO) at different time points after injury by fall armyworm, *Spodoptera frugiperda* in rice, *Oryza sativa*.

Effect	48 h		72 h				12 d			
	Phenolics ^§^		POD ^^^		PPO ^^^		POD ^§^		PPO ^§^	
	F	*p*	F	*p*	F	*p*	F	*p*	F	*p*
Si	0.84	0.373	0.19	0.668	0.80	0.385	1.04	0.324	1.01	0.331
AMF	4.50	0.050	3.65	0.075	0.55	0.472	2.66	0.123	13.44	**0.002**
Injury	0.09	0.770	0.75	0.401	6.46	**0.022**	2.63	0.124	1.40	0.254
Si * AMF	0.03	0.873	0.32	0.579	0.03	0.858	0.43	0.522	0.00	0.995
Si * Injury	3.42	0.083	1.25	0.281	0.04	0.853	3.58	0.077	3.98	0.063
AMF * Injury	2.73	0.118	7.25	**0.017**	29.75	**0.000**	0.97	0.340	2.14	0.163
Si * AMF * Injury	0.20	0.663	0.24	0.630	0.18	0.678	1.14	0.301	1.98	0.178

§ degrees of freedom: numerator and denominator degrees of freedom are 1 and 16, respectively, for all effects. ^ degrees of freedom: numerator and denominator degrees of freedom are 1 and 15, respectively, for all effects. * indicates interaction between the main effects. Peroxidase and polyphenol oxidase activity is reported as change in absorbance/min/g of fresh weight of leaf tissue. Plant phenolics are expressed as nmoles ferulic acid equivalents per mg of fresh weight of leaf tissue. F values of F in three-way ANOVA; *p* values of *p* in three-way ANOVA. Data of peroxidase at 12 day timepoint were square-root transformed for analysis. *p* values in bold indicate a significant main/interaction effect (*p* < 0.05).
